# Meta-modeling on detailed geography for accurate prediction of invasive alien species dispersal

**DOI:** 10.1038/s41598-019-52763-9

**Published:** 2019-11-07

**Authors:** Nick Pepper, Luca Gerardo-Giorda, Francesco Montomoli

**Affiliations:** 1 UQLab, Department of Aeronautics, Imperial College London, SW7 2AZ London, UK; 2 BCAM – Basque Center for Applied Mathematics, Bilbao, Spain

**Keywords:** Invasive species, Theoretical ecology

## Abstract

Invasive species are recognized as a significant threat to biodiversity. The mathematical modeling of their spatio-temporal dynamics can provide significant help to environmental managers in devising suitable control strategies. Several mathematical approaches have been proposed in recent decades to efficiently model the dispersal of invasive species. Relying on the assumption that the dispersal of an individual is random, but the density of individuals at the scale of the population can be considered smooth, reaction-diffusion models are a good trade-off between model complexity and flexibility for use in different situations. In this paper we present a continuous reaction-diffusion model coupled with arbitrary Polynomial Chaos (aPC) to assess the impact of uncertainties in the model parameters. We show how the finite elements framework is well-suited to handle important landscape heterogeneities as elevation and the complex geometries associated with the boundaries of an actual geographical region. We demonstrate the main capabilities of the proposed coupled model by assessing the uncertainties in the invasion of an alien species invading the Basque Country region in Northern Spain.

## Introduction

The spread of invasive species is responsible for severe ecological damage and economic losses and is recognised as a significant threat to biodiversity^1,2^. Its management is a challenging problem due to the uncertainty surrounding the species characteristics and the usually limited resources allocated to control. Firstly, measures to protect the native species of a region from invasive species are costly for the local government. For example, the Basque water agency (URA) budgets an average of €1.4 million a year for activities related to the elimination of invasive species and the restoration of habitats. In addition to this figure, the Biodiversity Strategy of the Basque Autonomous Community 2030 calls for €1,119,470 to be spent on the protection and restoration of habitats in 2019 alone^3^. Moreover, the management often starts long after the appearance of the invasive species, when the ecological damage is already visible and the species well-established, taking an additional financial toll. Control strategies may aim at extirpation or rely on forcing a component Allee effect by bringing the population below a threshold^4^, for instance by increasing the abundance of predators, by culling or inducing habitat fragmentation^5^.

Another important consideration is the need to account for the temporal dynamics of the invader population. Managers have to optimize their choices not only for the present, but must also take into account possible future scenarios. The introduction of *cane toad* to Australia from Hawaii in 1935 in an attempt to control the native grey-backed *cane beetle* and its subsequent, uncontrolled, spread throughout the continent is a cautionary tale in this respect^6^. On the other hand, if the understanding of population persistence and competitive outcomes can provide long-term information, then short-term predictions of spread are often more important in the management of invasive species as fast responses can potentially save public administrations sizeable amounts of money^7^. Optimal management strategies generally rely on the coupling of biological models with optimization procedures and are reviewed in Epanchin-Niell and Hastings^8^. Within such a framework, the modeling of invasive species and the dynamics of their dispersal can be of great help to environment conservation agents and policy makers.

A number of works have appeared in recent decades which have aimed to mathematically model invasive species at different levels of accuracy. A popular technique in devising predictive models has been Spatially Explicit Population dynamics Models (SEPMs). Introduced by Rushton and collaborators in 1997^9^ in the study of red and grey squirrel distribution at landscape scale, SEPMs are models that combine GIS data and a population dynamics approach on separated habitat blocks. These models have been used quite effectively to study the invasive expansion^10^ and control^11^ of the grey squirrel in northern Italy. The major drawback of SEPMs is that their performance is sensitive to the accuracy of the estimates of the life-history parameter input. As a consequence, SEPMs can only be used for species for which detailed data of population parameters (fecundity, mortality, dispersal distance, density) in different types of habitat are available. Expanding on the ideas of SEPMs, Jones *et al*.^12^ recently used a Gis-Grass based deterministic model with stochastic rates^13^ to study control strategies to protect native red squirrels from invasive grey squirrels on the island of Anglesey in the UK. Catford *et al*.^14^ used boosted regression trees to identify areas within a region of interest that are the most susceptible to invasion by non-native plant species. The model is not species specific and uses a set of 18 uncorrelated variables to classify the most vulnerable regions, which can be prioritized in conservation efforts. Gassó *et al*.^15^ studied the distributions of native and invasive plant species in Catalonia, Spain and found that human influence on the landscape had a greater influence on the species richness of the invasive species than climatic variables. Campos *et al*.^16^ used a statistical approach on patches to investigate the effect of environmental factors on the invasion patterns of 89 invasive plants species in the Basque Country, concluding that the spatial structure of alien plant invasions in the region are strongly environmentally induced. A common trait of these works is that the impact of geography on the distribution of invasive species is recognised, even if the exact nature of the complex link between the landscape and the abundance of an invasive species is not completely understood^17^.

Accounting for the heterogeneities of the landscape in invasive species modeling is not a trivial task. Forests, plains and mountains present different levels of hospitability to the invasive species, whilst strong natural barriers such as motorways, lakes and major waterways can reduce, if not stop completely, the spread of the species^18^. Different approaches have been used in the literature to cope with the spatial component of heterogeneities, from data-driven statistical modeling to deterministic systems of differential equations. Lohr *et al*.^19^ investigated the effect of various land management options on local biodiversity via a system of ordinary differential equations for the abundance of two competing species, one local and one invasive, on an island. Although able to account for several important factors such as growth rate, carrying capacity, species interaction, efficacy and cost of management, their method is spatially lumped on each type of habitat. While on the one hand such a model can be very effective in the management of an already established population, it lacks the spatial accuracy required to predict an invasion and thus is of limited use in devising early interventional strategies.

Meier *et al*.^20^ combined species distribution models^21^ (SDMs) with population spread models^22^ (models which cover meta-population dynamics) to evaluate the effectiveness of control actions on three widespread invasive alien plants for different spending scenarios and management goals in a complex landscape on the Swiss plateau. They showed how control actions under a restricted budget are more effective if spatially prioritized and, at the same time, that applying intensive control at early stages generally increased the effectiveness of control. In a recent paper, Maciel and Lutscher^23^ studied the coexistence of two competitive species in dependence of habitat preference, diffusion rates and carrying capacity. In order to perform their analysis, based on homogenization techniques and a multi-scale approach, they needed to consider an idealized situation where the two species are very similar and located in a one-dimensional, patchy, infinite landscape where the distribution of habitat patches is periodical and alternates between strongly favourable and mildly favourable to the species. As a consequence, although this approach provides interesting insight on the competing dynamics, it cannot be immediately translated to a realistic landscape.

Since observational data is in general collected according to the geopolitical structure of a region, the intuitive idea behind the works presented above is to subdivide the region of interest into discrete units either geopolitically or territorially. A geopolitical subdivision follows administrative subdivisions at various levels such as at the levels of townships, counties or provinces, whilst a territorial subdivision divides the landscape into regular territorial patches and considers the species movements from one unit to another. Geopolitical decompositions have been used extensively in epidemiology, for example in Smith *et al*.^24^ where the spatial propagation of raccoon rabies in Conneticut, USA is modelled. Although a priori well adapted to observational data, a geopolitical approach suffers from significant limitations when considering wildlife. On the one hand, animals do not in general move at the geopolitical scale, nor do their movements fit the shape of geopolitical units. On the other hand, plants do not move and their diffusion is driven by the dispersal of seeds that can be transported by climate agents such as wind or rivers, but in general do not move very far from the plant that generated them. As a consequence, several scientists have applied continuous reaction-diffusion models based on Fisher’s equation^25^ to problems in ecology. Reaction-diffusion models rely on the implicit assumption that the dispersal of an individual is random but at the scale of the population the density of individuals is smooth and the invasion front is somewhat regular^26^. Recently, Bonneau *et al*.^27^ applied a reaction diffusion model to simulate the effect of management efforts on the distribution of Burmese pythons in the Everglades (Florida, USA). The reaction-diffusion method was shown to yield better prediction accuracy than the more established Constant Effort Harvesting model. A limitation of the study is that the region of interest was treated as an isolated environment with zero flux boundary conditions imposed. This is a common feature to many works (not only in reaction-diffusion models), where the considered environment is either an island or a region bounded by impenetrable borders and where the effect of migration from neighboring domains is not considered^12,19,28–30^.

Although reaction-diffusion models are a good trade-off between model complexity and flexibility for use in different situations, their limited application resides in the technique used in their numerical approximation. Common reaction-diffusion models are approximated in space by means of finite differences methods (FD, see, e.g.^31^). The natural framework of FD requires the use of a Cartesian discretization grid. As the latter cannot adapt to the complex geometries that represent the boundaries of a real geographical region, researchers using reaction-diffusion models have limited themselves to either idealized geometries (one dimensional intervals^23^ or rectangles^27,32^) or covered the land under study through lattices composed of rectangular patches with sizes that are too large to cover smaller heterogeneities. As an example, Hooten and Wikle^33^ used, in a Bayesian setting, a finite difference approach on a lattice to approximate the latent process for the logarithm of the intensity of a Poisson process describing the abundance of the Eurasian Collared dove in the South East of the USA. In his PhD Thesis, Arab^34^ suggested an extension to this approach by using finite elements in the approximation of the latent process, but both his derivation and implementation never went past a one-dimensional interval.

In this paper, we consider a continuous, time-dependent, nonlinear reaction-diffusion model for the species density that arises from a generalization of the Fisher equation. Following an idea introduced in Keller *et al*.^35^, in which the spread of rabies among raccoons in New York State was studied, landscape heterogeneities are accounted for by including in the computational domain the significant geographical features of the area by acting directly on the coefficients of the model that are both spatially and temporally dependent. In particular, we show how the model can account for elevation in a natural way. Elevation is a major trait in landscape heterogeneity, pretty much overlooked in the literature, despite the fact that quite often species’ habitats are bounded by it^18^. Moreover, recent studies focusing on the impact of climate change in modifying the habitat of species have found that increased temperature and changes in humidity have rendered higher altitudes suddenly hospitable for new species that might pose a threat to indigenous ones (see e.g.^22,36–39^). From this perspective, the simplicity with which the model proposed here is able to account for elevation can become a major asset. The reaction-diffusion model is numerically approximated by the finite element method, which is able to treat arbitrarily shaped boundaries, like the ones of a geographical region^40^. Moreover, finite elements can easily be employed on adaptive grids that feature finer resolution in specific regions of interest, for instance co-localized with peculiar landscape characteristics.

For a tool aimed at supporting management strategy in environmental conservation it is necessary to provide not only responses on suitable quantities of interest commonly used in the decision process, but also confidence intervals. To cope with how the uncertainty in the data and the characteristics of the species are propagated by the proposed model, we couple the reaction-diffusion equation with arbitrary Polynomial Chaos (aPC, see, e.g.^41^). Quantifying uncertainty is important for predictive models, and may become paramount if conservation agencies are faced with the threat from a species about which very little is known. Arbitrary Polynomial Chaos, being very efficient in its handling of scarce data, appears a natural choice.

This paper is organized as follows. In Section 2 we introduce a deterministic continuous advection-diffusion-reaction term model for the dispersal of an invasive species in an heterogeneous environment; describe the way it accounts for geographical characteristics and present the simulation protocol. In Section 3 the deterministic model is demonstrated by its application to an alien species invading the Basque Country region in Northern Spain. Section 4 introduces the arbitrary Polynomial Chaos (aPC) method used to propagate parametric uncertainties through the model. The method is then applied to assess uncertainty on the test case introduced in Section 3 using synthetic data.

## Methods

### A general distributed model for species dynamics

The spread of an invasive species in a region denoted by $\Omega \subset {{\mathbb{R}}}^{2}$ can be modeled by a modified Fisher’s equation for the density $u(\overrightarrow{x},t)$ of individuals in location $\overrightarrow{x}$ at time *t* (see^42^ for an introduction to diffusion in ecological problems). In the more general form the model is a nonlinear advection-diffusion-reaction equation of parabolic type:

1 $${{\rm{\partial }}}_{t}u-{\rm{d}}{\rm{i}}{\rm{v}}\,({\boldsymbol{\nu }}(\overrightarrow{x},t){\rm{\nabla }}u)+{\bf{b}}(\overrightarrow{x},t)\cdot {\rm{\nabla }}u+u(u-\gamma (\overrightarrow{x},t))=f(\overrightarrow{x},t)\,{\rm{i}}{\rm{n}}\,\Omega \times [0,T].$$

In Eq. (1) the diffusion coefficient $\nu (\overrightarrow{x},t)$ accounts for the static landscape heterogeneities such as inhospitable regions and the presence of natural or artificial barriers within the computational domain. Examples of such barriers include major waterways and roads. The carrying capacity $\gamma (\overrightarrow{x},t)$ expresses the birth and death rates of the species at a given spatial and temporal location. Their spatial dependence allows the inclusion of peculiar landscape characteristics in terms of the classification of the terrain (town, meadow, rock, wood, elevation). The convective term ${\bf{b}}(\overrightarrow{x},t)\cdot \nabla u$ allows for transport effects within the region to be modeled. In the case of terrestrial amphibious animals, the transport effects are associated with the presence of rivers that can act both as an accelerator or as a contrasting agent according to their flow direction with respect to the direction of the propagation front; outside of waterways the transport effects vanish. The temporal dependence of the coefficients allows for various types of seasonality: variations in the level of water and flow velocity of rivers, breeding for animals or sprouting for plants. Considering seasonality can be very important since, as shown by Meier *et al*.^20^, the most effective spatial treatments against invasive species prioritize small populations in the case of the seasonal species and large populations in the case of the perennial species.

Equation (1) is completed by a suitable initial value ${u}^{0}(\overrightarrow{x})$ and boundary conditions on ∂Ω^43^. In general, homogeneous Neumann boundary conditions are used to model an isolated environment, but other boundary conditions can be considered as well: a homogeneous Dirichlet boundary condition would model an hostile environment, while a Robin boundary condition would model migratory dynamics^27,42^. Finally, let us point out that long distance dispersal could be included in the model by adding a stochastic term to the reaction-diffusion equation, but this goes beyond the scope of this paper.

### Geographical accuracy of the model

#### Elevation

A geographically detailed description of the region of interest should not only include evident landscape heterogeneities such as rivers, lakes or urban areas, but also elevation. The latter can play a crucial role in characterising the hospitality of an area for a given species. Commonly both animal and plant species are suited to habitats in a bounded region of elevation and this aspect should be taken into account. The NASA Shuttle Radar Topography Mission (SRTM, February 11 to 22, 2000) produced a near-global covering of land on Earth, although limited to latitudes between 60S and 60N. Files containing the elevation data have been made publicly available (the elevation data has been collected in files available at http://dds.cr.usgs.gov/srtm/version2_1). Elevation values are integers (INT16 class): sea level values are 0, unknown values equal −32768 (there is no NaN for INT class). Each file corresponds to a tile of 1 × 1 degree of a square 1201 × 1201 grid, consisting of latitude, longitude, and elevation values (SRTM3 = 3 arc-seconds). For territories within the USA the tiles feature a higher resolution of 3601 × 3601 grid (SRTM1 = 1 arc-second). By using this data, a three dimensional grid may be generated for a given region of interest that lies between 60S and 60N. In what follows, we will denote by $z=z(\overrightarrow{x})$ the elevation at point $\overrightarrow{x}\in \Omega$.

#### Extended heterogeneities and carrying capacity

Strong, extended heterogeneities may significantly affect the dynamics of the dispersal across a whole region. To cope with such features we take advantage of the temporal and spatial dependence of the carrying capacity and the diffusion coefficient in Eq. (1). In general, we consider the carrying capacity to be naturally dependent upon the elevation of the ground, however, different levels of hospitality can occur at a given elevation due to the landscape heterogeneities and such features can be taken into account. Less hospitable regions are modeled by lowering the carrying capacity to reduce the population density at equilibrium (see also^23^). The diffusion coefficient is also reduced to minimize the dispersal towards such inhospitable regions. Lakes are included in the model by setting the carrying capacity to 0 in their interior.

#### Treatment of boundary condition artifacts

Geographical regions are usually characterized by fairly irregular boundaries. Few approaches in the literature use real geographical boundaries or impose no-flux boundary conditions in the cases that do, modeling the region of interest as an isolated environment. Whilst this may be a reasonable assumption if the region of interest is an island, in the case of a territory continuous with surrounding areas the choice of restricting the modeling to the actual region of interest could trigger unrealistic features in the vicinity of its boundary. To avoid such an effect, a computational domain larger than the region of interest is considered. This approach is commonly used in Computational Fluid Dynamics (CFD) simulations in aeronautics^44^: if the computational domain is sufficiently large, a zero flux boundary condition can be imposed without impairing the simulation in the region of interest.

### Numerical approximation

The finite element method (FEM, see^40^ for an introduction to the subject) is particularly suitable for complex geometries like the ones represented by real geographical regions. We thus discretize Eq. (1) in space by means of finite elements, while a classical finite difference discretization is carried out for the time discretization.

#### Spatial discretization

For a regular triangulation (also called mesh) of the domain Ω, a finite element space of order *k* consists of globally continuous functions that are locally a polynomial of degree at most *k* on every triangle of the mesh. Each function of a finite element space can be represented as a linear combination of a suitable finite basis. In the numerical simulations presented in this paper we will use first order elements. Although higher order polynomials can be considered^40^, linear ones feature a sufficient level of accuracy for the applications being considering here. A generic element of the first order basis, denoted with ${\phi }_{j}(\overrightarrow{x})$ (*j* = 1, …, *N*, *N* being the total number of mesh points), is the piecewise linear function equal to 1 on the *j*^*th*^ node of the mesh and equal to 0 in all the other nodes. The finite element approximation of the solution of Eq. (1) is given by

2 $${\overrightarrow{u}}_{h}(\overrightarrow{x},t)=\mathop{\sum }\limits_{j=1}^{N}{u}_{j}(t){\phi }_{j}(\overrightarrow{x}).$$

The unknown time-dependent vector $\overrightarrow{u}(t)={[{u}_{1}(t),\ldots ,{u}_{N}(t)]}^{T}$ solves the *N*-dimensional nonlinear ordinary differential system

3 $${\rm{M}}\,\frac{d\overrightarrow{u}(t)}{dt}+{\rm{A}}\,\overrightarrow{u}(t)+{\rm{B}}\,\overrightarrow{u}(t)= {\mathcal F} (\overrightarrow{u}(t)),$$

where M, A, and B are the mass, stiffness and transport matrices, whose (*i*, *j*)-th entries are

4 $${{\rm{M}}}_{ij}=\mathop{\int }\limits_{\Omega }\,{\phi }_{j}{\phi }_{i}\,d\overrightarrow{x},\,{{\rm{A}}}_{ij}=\mathop{\int }\limits_{\Omega }\,(\nu (\overrightarrow{x})\nabla {\phi }_{j})\nabla {\phi }_{i}\,d\overrightarrow{x},\,{{\rm{B}}}_{ij}=\mathop{\int }\limits_{\Omega }\,(\overrightarrow{b}(\overrightarrow{x})\cdot \nabla {\phi }_{j}){\phi }_{i}\,d\overrightarrow{x},$$

while ${\mathscr{F}}(\overrightarrow{u}(t))=\mathop{\int }\limits_{\Omega }\,(\gamma (\overrightarrow{x},t)-{\overrightarrow{u}}_{h}(t))\,{\overrightarrow{u}}_{h}\cdot {\phi }_{i}\,d\overrightarrow{x}$ is the discretization of the nonlinear term. All of the above integrals are computed by means of suitable quadrature rules.

#### Time discretization

Let Δ*t* be a time step and *t*^*n*^ = *n*Δ*t* be the discretization of the time interval under study. We denote by ${{\bf{u}}}^{n}={[{{\bf{u}}}_{l}({t}^{n})]}_{l=1,\ldots ,N}$ the vector of the nodal values at time *t*^*n*^. The incremental ratio (**u**^*n*+1^ − **u**^*n*^)/Δ*t* is an approximation of the time derivative either in *t*^*n*+1^ or *t*^*n*^, with an associated numerical error proportional to Δ*t*. As a good trade-off between numerical stability and computational efficiency, Eq. (3) is advanced in time by a mixed implicit/explicit (IMEX) approximation scheme, where the stiffness and the transport are treated implicitly while the nonlinear term is treated explicitly. Knowing the approximation **u**^*n*^ of the solution at time step *t*^*n*^, the solution at time step *t*^*n*+1^ is obtained by solving

5 $$({\rm{M}}+\Delta t\,{\rm{A}}+\Delta t\,{\rm{B}}){{\bf{u}}}^{n+1}={\rm{M}}{{\bf{u}}}^{n}+\Delta t\,{\rm{F}}({{\bf{u}}}^{n}).$$

Equation (5) is a linear system of dimension *N*, where the right hand side is easily built since **u**^*n*^ is known. For a more extensive discussion and application of the IMEX method see^31^.

#### Simulation protocol

The numerical simulations of Eq. (1) are performed with a self-developed code in Matlab (MathWorks Inc., Natick, MA) with a uniform time step of Δ*t* = 0.1 months. Several techniques to efficiently solve linear systems like (5) are available based on iterative methods: at every time step we solve (5) with the conjugate gradient method, preconditioned by an incomplete Cholesky factorization (see, for instance the book by Y. Saad^45^). The Matlab source code is available as supplementary material to this paper.

#### Data driven mesh generation

The computational domain is discretized by a triangular mesh to exploit its higher accuracy (compared to a typical cartesian grid for finite differences approximations) in representing both complex boundaries and peculiar structures in the interior of the domain. Adaptive grids are used in order to harness the best approximation properties of the finite element method. These feature smaller triangles in the areas that require higher levels of accuracy in the landscape, like the region of interest and the heterogeneities of the landscape. The geographical details described in the previous sections can be effectively implemented on a mesh whose generation is outlined by the following procedure:

1. Identify the region of interest in the computational domain and its main geographical features (e.g. rivers, lakes, urban areas)
2. Generate an initial grid ${{\mathscr{T}}}_{0}$, uniformly refined over the region of interest.
3. Include elevation information from NASA SRTM data by interpolation on the grid ${{\mathscr{T}}}_{0}$.
4. Refine ${{\mathscr{T}}}_{0}$ in the surroundings of the main geographical features identified in step (1).
5. Implement a gradient-driven refinement of ${{\mathscr{T}}}_{0}$ where the elevation gradients are steeper (like along the sides of a valley).

## Basque Country Test Case

**Figure 1:**
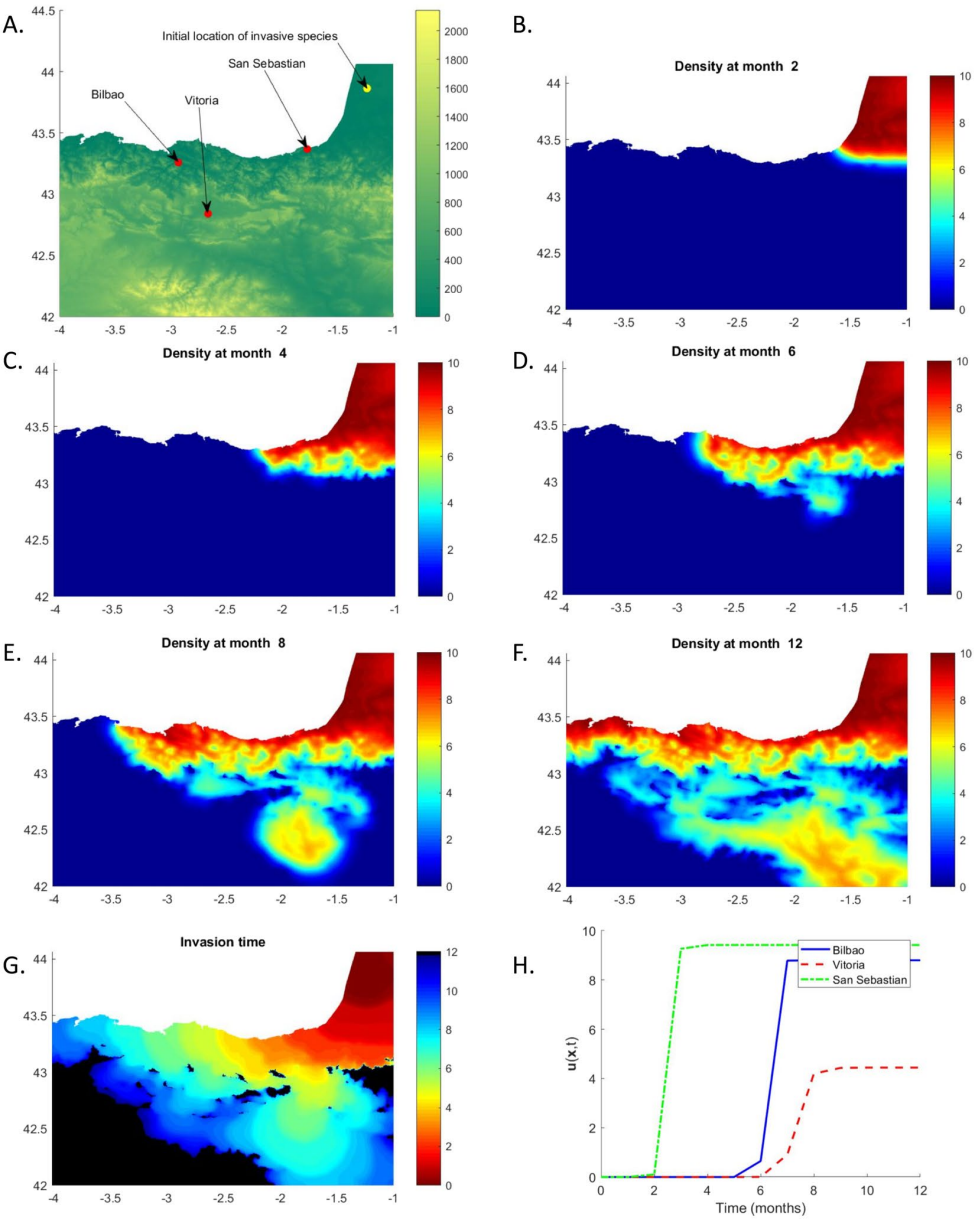
Basque Country test case. Panel (A) Computational domain with elevation, initial location of the invasive species and location of the three cities under study. Panel (B to F) Density of the invasive species at different times of the invasion process. The temporal dynamics highlights how extended heterogeneities due to the mountain ranges favors dispersal to the West. Panel (G) Invasion time in the whole computational domain. Black areas are inhospitable for the species and will not be invaded. Panel (H) Temporal dynamics of the population density $u(\overrightarrow{x},t)$ for Bilbao (solid blue), Vitoria (dashed red) and San Sebastian (dot-dashed green).

To illustrate the characteristics of our method, we simulate the dispersal of an invasive species in the territory of the Basque Country in Northern Spain. The region itself is an excellent testbed, as the heterogeneity of its landscape encompasses various types of environment from the sea shores of the Bay of Biscay along the Atlantic Ocean to the mountainous region in the interior, to the higher peaks of the nearby Pyrenees range. The computational domain Ω is the region comprised between 4°W and 1°W in Longitude, and between 42°N and the Bay of Biscay (or 44°2′N) in Latitude (see Fig. 1, panel A). The domain Ω contains the region of interest and is triangulated according to Steps (1–5) described in the previous Section. The mesh consists of 78,832 points and 155,478 triangular elements. Note that throughout the rest of the paper co-ordinates will be expressed in Longitude and Latitude.

In the simulated scenario the invasive species is initially absent from the region of interest and is concentrated in a circle of radius 0.5 centred in the Aquitaine region in south-western France (−1.2349, 43.8631). From the perspective of environmental conservation, the natural interest is in the population density and speed of propagation. Some more specific quantities of interest may be the arrival time to specific, sensitive locations (like a natural reserve). As an example we consider the three major cities in Basque Country: Vitoria, Bilbao and San Sebastian. Their locations and the initial position of the invasive species are highlighted in Fig. 1, panel A, that also shows the elevation of the region. As this is an illustrative case, for numerical simplicity the impact of rivers was assumed to be negligible compared to the extended geographical heterogeneities induced by the mountain ranges. We assume a constant diffusion coefficient *ν* = 5 × 10^−4^ deg^2^ years^−1^ while the carrying capacity for the species is assumed to depend solely on elevation:

6 $$\gamma (\overrightarrow{x},t)=(10-\frac{z(\overrightarrow{x})}{100}).$$

The carrying capacity is thus maximum at sea level and is linearly decreasing with elevation. The species is assumed to be unable to survive at elevations greater than 1000 meters above sea level. Results of the dispersal simulation are shown in Fig. 1, panels B to F. As expected, higher densities are observed along the coastline. At the same time, the impact of elevation is evident. In particular, the barrier effect played by the Pyréneens ridge running along the 43^rd^ parallel significantly affects the dispersal and forces the species to move west (panel B and C) until the presence of a valley in the N-S direction allows the species to move south as well (panel D and E). Panel G in Fig. 1 shows the arrival time (*t*_*arrival*_) of the species in the whole computational domain: *t*_*arrival*_ is defined in any point $\overrightarrow{x}$ of the domain Ω as the time at which the population density reaches 1 i.e. $u(\overrightarrow{x},{t}_{arrival})=1$. Black areas are inhospitable for the species due to their elevation and will not be invaded. Finally, panel H in Fig. 1 shows the temporal evolution of the population density in the three cities of interest. Naturally, San Sebastian is the first city reached by the species. Nevertheless, Bilbao is reached before Vitoria, despite the second one being closer to the initial distribution of the invasive species. Finally, the population density in Vitoria is significantly lower than in Bilbao and San Sebastian due to Vitoria’s higher elevation (as can be inferred from Fig. 1, panel A).

## Uncertainty Quantification

### Propagating model uncertainty using arbitrary polynomial chaos

As the invasive species dispersal model is intended to be an aide in decision-taking processes for policymakers, it is necessary to quantify the model uncertainties in order for its predictions to be trustworthy. It is likely that the exact values of the coefficients in Eq. (1) are not known precisely but perhaps are known in distribution form. An arbitrary Polynomial Chaos (aPC) method is used to quantify how the variation in the values of the model coefficients affect the predictions of the spread of the invasive species. A summary of data driven aPC is provided here, for a fuller description the reader is referred to the work of Oladyshkin^41^.

Data driven aPC has two advantages over the more traditional Monte Carlo sampling technique: firstly, it is far less computationally expensive than Monte Carlo sampling and secondly, the method is able to handle scarce data. The computational efficiency of the method comes from the construction of a sparse grid for sampling using Smolyak’s rule. For each input distribution the optimal Gaussian quadrature points and weights are computed using the distributions’ statistical moments. Repeating this process for all *N*_*d*_ input distributions will produce a sequence of one dimensional quadrature rules ${\{{U}^{{i}_{j}}\}}_{j=1\ldots {N}_{d}}$ with collocation points ${\xi }_{k}^{{i}_{j}}$ and weights ${\omega }_{k}^{{i}_{j}}$:

7 $${U}^{{i}_{j}}=\mathop{\sum }\limits_{k=1}^{{m}_{{i}_{j}}}w({\xi }_{k}^{{i}_{j}}){\omega }_{k}^{{i}_{j}},$$

where ${m}_{{i}_{j}}j\in \{1,\ldots {N}_{u}\}$ is the maximum adaptive order for each quadrature rule and $w({\xi }_{k}^{{i}_{j}})$ refers to a model evaluation at the collocation point ${\xi }_{k}^{{i}_{j}}$. Through the application of Smolyak’s rule it is possible to find a sparse grid to sample over:

8 $${A}_{s}=\sum _{l+1\le |i|\le l+{N}_{u}}{(l-1)}^{l+{N}_{u}-|i|}(\begin{array}{c}{N}_{u}-1\\ l+{N}_{u}-|i|\end{array}){\otimes }_{k=1}^{{N}_{U}}{U}^{i}.$$

Increasing the level of the quadrature increases the accuracy of the result as more points are added to the grid, but at increased computational cost. Having obtained a sparse grid with *N*_*sp*_ points from Smolyak’s rule the data driven arbitrary Polynomial Chaos representation of the model output is approximated as:

9 $$w(\xi )\approx \mathop{\sum }\limits_{k=1}^{{N}_{sp}}\,{\alpha }_{k}{\psi }_{k}(\xi ),\,{\rm{with}}\,{\alpha }_{k}=\frac{{\sum }_{i=1}^{{N}_{sp}}\,w({\eta }_{i}){\psi }_{k}({\eta }_{i}){\theta }_{i}}{{\sum }_{i=1}^{{N}_{sp}}\,{\psi }_{k}({\eta }_{i}){\theta }_{i}},$$

where *ψ*_*k*_ refers to the *k*^*th*^ order polynomial in a family of orthogonal polynomials, while *η*_*i*_ and *θ*_*i*_ represent the sparse grid collocation points and weights. Through Monte Carlo simulation of the PCE representation of the model output it is possible to build an output probability distribution function (PDF) for the model.

### Uncertainty analysis for the Basque Country test case

An uncertainty analysis was performed for the Basque Country test case. As an illustrative example we explored uncertainty in both diffusion (affecting propagation, thus the speed of the invasion) and carrying capacity (playing a key role in the settling of the species in a given location). To this end, we considered a modified version of the carrying capacity, scaled by a random parameter *α*:

10 $$\gamma (\overrightarrow{x},t)=\alpha (10-\frac{z(\overrightarrow{x})}{100}).$$

**Figure 2:**
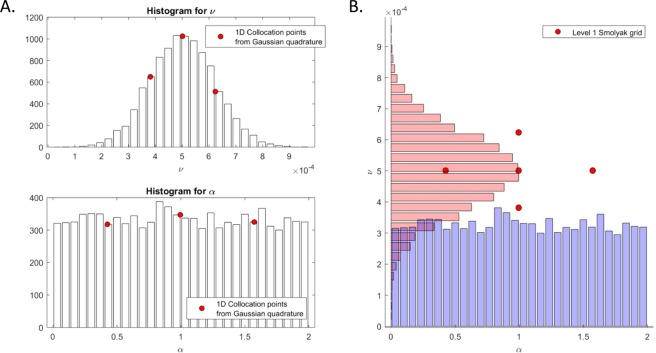
Panel (A) Histograms for the uncertain parameters *ν* and *α*, and the 1D collocation points for the input distributions. Panel (B) The sampling grid calculated through application of Smolyak’s rule at level 1.

**Figure 3:**
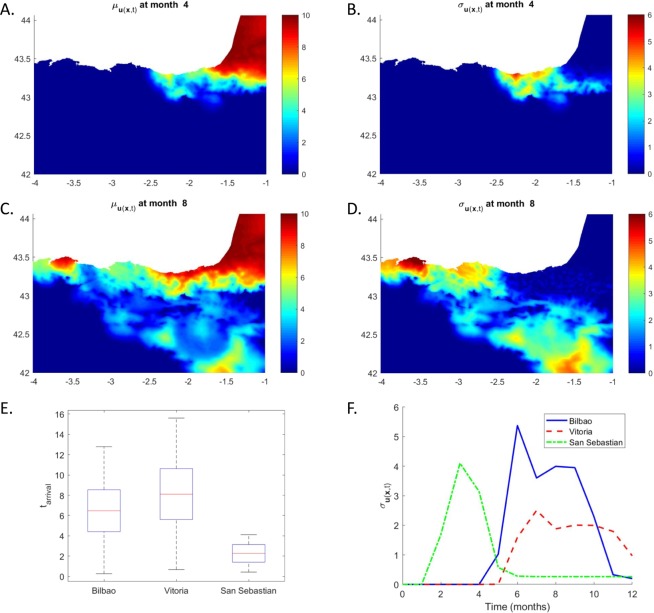
Uncertainty analysis for the Basque country test case. Panel (A to D) Temporal dynamics of mean and standard deviation of the population density in the whole computational domain highlights the higher level of uncertainty in the surrounding of the wavefront. Panel (E) Uncertainty in arrival time in Bilbao, Vitoria and San Sebastian. Panel (F) Temporal dynamics of the standard deviation for the population density in Bilbao (solid blue), Vitoria (dashed red) and San Sebastian (dot-dashed green) shows how uncertainty drops significantly in the wake of the propagation wavefront.

The uncertain parameters are thus the diffusion coefficient, *ν*, and the scaling factor *α*. Histograms for the two uncertain parameters were generated synthetically and are shown in panel A of Fig. 2. The histogram for *ν* was generated from a normal distribution *N*(5 × 10^−4^, 1.2 × 10^−4^), while *α* was sampled from the uniform distribution *U*(0, 2). Having generated synthetic histograms for *ν* and *α*, data driven aPC was used to propagate the uncertainties in these parameters through the invasive species model. The aPC technique was implemented in self-developed code following the procedure described in^46^. The simulation code can be made available by contacting *f*.*montomoli@imperial*.*ac*.*uk*. Panel A in Fig. 2 shows also the locations of the 1D collocation points for the two input distributions. Having found the collocation points and weights from the data, Smolyak’s rule is applied to generate a level 1 grid. In this case the resulting grid consists of 5 points, shown in panel B of Fig. 2. The invasive species model was evaluated at each of the points and based on the results, PDFs of $\overrightarrow{u}(\overrightarrow{x},t)$ were calculated, at each time step, in the whole computational domain. Figure 3 collects the results. Panels A to D show the mean (*μ*, A and C) and the standard deviation (*σ*, B and D) of the PDFs on the whole region at different time steps. The bottom row of Fig. 3 show the results for Bilbao, Vitoria and San Sebastian. Panel E represent uncertainty in the arrival times of the species in the three cities, while panel F shows the temporal dynamics of the standard deviation of the population density. Panels B, D, and F highlight how uncertainty is mainly concentrated in the vicinity of the propagation front and drops significantly in its wake.

## Conclusions

Predictive modeling of the spatio-temporal spread of an invasive species is a very useful tool to help environmental conservation policymakers in their decision-making process for devising appropriate countermeasures. As model parameters for invasive species are often inferred form field measurements, they are imbued with uncertainty. If the species is poorly understood then the uncertainty may be so significant that it affects the reliability of the model predictions. A continuous reaction-diffusion model for the population density of an invasive species has been presented in this paper, which has been coupled with an arbitrary Polynomial Chaos (aPC) method to assess how the uncertainty of poorly known parameters affects the predictions of the deterministic model. The coupled model has several strengths. Firstly, aPC is far less computationally expensive compared to a classical Monte Carlo sampling method. More importantly, aPC is able to handle scarce data and in this respect the proposed model is far more effective than SEPMs (which require very detailed data about the population parameters to achieve good performance)^9–12^, and can be employed to estimate the initial stages of the invasion. Secondly, accurate geographical modeling can be easily included in the proposed framework since the continuous reaction-diffusion model has been implemented using a Finite Element formulation. Such an approach allows the treatment of general geometries like a real geography and its actual boundaries. At the same time, it allows landscape heterogeneities to be treated in a natural way, differentiating it from other works in the literature which, relying on Finite Differences, have lacked spatial accuracy by considering species dispersal on simplified or idealised landscapes (either one-dimensional or cartesian)^23,27,32^. The deterministic model presented here is equipped with a data-driven mesh generator that allows added refinement in the region of interest and in heterogeneities in the landscape. Finally, the individual runs of the deterministic model needed to set up the aPC procedure are not computationally demanding and can be computed on a laptop in few minutes. As a proof of concept, in this paper we demonstrate the practical applicability of the proposed method by quantifying the uncertainty in the spread of a generic invasive population in the Basque Country area in northern Spain.

For the sake of simplicity in presentation the method has been described here in basic form, but it can be easily extended in a number of ways. For instance, in our proof of concept we showed how elevation can be easily included and the carrying capacity was dependent solely on it. While the elevation is an important^18,22,36–39^ and, as has been discussed, often overlooked factor in species dispersal it is of course not the only factor. In practice the carrying capacity may be a function of a number of environmental variables. The spatial dependence of the model coefficients allows other characteristics concerning hospitality to be easily incorporated. As an example, the landscape can be differentiated between meadows, forests, urban areas and mountains, whose geographical distribution can be obtained from GIS data^11^. Moreover, the temporal dependence of the coefficients allows for temporal variations to be accounted for in the dynamics. For instance seasonality could be modeled by periodizing relevant coefficients in the reaction-diffusion equation. Another important aspect to underline is that, for illustrative purposes, this paper considered the very simple situation of a single species invading an empty environment. However, any ecological model of competition based on systems of ordinary differential equations (like the one in Lohr *et al*.^19^, for instance) could be easily included in our framework. The resulting model would be a system of partial differential equations of reaction-diffusion type like the one studied in one spatial dimension by Maciel and Lutscher^23^.

The presented model has some limitations as well. Firstly, although aPC is very efficient in handling scarce data, a preliminary parameter estimation for the model coefficients is needed. As population data are normally available at geopolitical or territorial levels, particular care would be required to translate them at a spatially continuous level. Secondly, although the proposed coupled model provides a probability distribution for relevant quantities of interest in species invasion, it lacks the ability of reproducing peculiar dynamics such as the stochastic fade-out characteristic of individual based models in epidemiology^13^.

## Supplementary information

Deterministic model code
